# Bacterial diversity in faeces from polar bear (*Ursus maritimus*) in Arctic Svalbard

**DOI:** 10.1186/1471-2180-10-10

**Published:** 2010-01-14

**Authors:** Trine Glad, Pål Bernhardsen, Kaare M Nielsen, Lorenzo Brusetti, Magnus Andersen, Jon Aars, Monica A Sundset

**Affiliations:** 1 Department of Pharmacy, University of Tromsø, 9037 Tromsø, Norway; 2 Department of Arctic Biology and Institute of Medical Biology, University of Tromsø, 9037 Tromsø, Norway; 3 Faculty of Science and Technology, Free University of Bozen/Bolzano, Bolzano, Italy; 4 Norwegian Polar Institute, 9296 Tromsø, Norway

## Abstract

**Background:**

Polar bears (*Ursus maritimus*) are major predators in the Arctic marine ecosystem, feeding mainly on seals, and living closely associated with sea ice. Little is known of their gut microbial ecology and the main purpose of this study was to investigate the microbial diversity in faeces of polar bears in Svalbard, Norway (74-81°N, 10-33°E). In addition the level of *bla*_TEM _alleles, encoding ampicillin resistance (amp^r^) were determined. In total, ten samples were collected from ten individual bears, rectum swabs from five individuals in 2004 and faeces samples from five individuals in 2006.

**Results:**

A 16S rRNA gene clone library was constructed, and all sequences obtained from 161 clones showed affiliation with the phylum *Firmicutes*, with 160 sequences identified as *Clostridiales *and one sequence identified as unclassified *Firmicutes*. The majority of the sequences (70%) were affiliated with the genus *Clostridium*. Aerobic heterotrophic cell counts on chocolate agar ranged between 5.0 × 10^4 ^to 1.6 × 10^6 ^colony forming units (cfu)/ml for the rectum swabs and 4.0 × 10^3 ^to 1.0 × 10^5 ^cfu/g for the faeces samples. The proportion of amp^r ^bacteria ranged from 0% to 44%. All of 144 randomly selected amp^r ^isolates tested positive for enzymatic β-lactamase activity. Three % of the amp^r ^isolates from the rectal samples yielded positive results when screened for the presence of *bla*_TEM _genes by PCR. *Bla*_TEM _alleles were also detected by PCR in two out of three total faecal DNA samples from polar bears.

**Conclusion:**

The bacterial diversity in faeces from polar bears in their natural environment in Svalbard is low compared to other animal species, with all obtained clones affiliating to *Firmicutes*. Furthermore, only low levels of *bla*_TEM _alleles were detected in contrast to their increasing prevalence in some clinical and commensal bacterial populations.

## Background

The gastrointestinal microbiota of animals play an important role in the maintenance of health and modulation of disease. Previously, ecosystems have been characterized using microbiological methods based on culturing and phenotypic analysis of the isolates. Since the growth requirements of many bacteria are unknown, most of the gastrointestinal bacteria remain uncultivated. Molecular studies, avoiding the cultivation bias, yield more detailed insight into the diversity and characteristics of the intestinal ecosystems. Most cultivation independent studies have been conducted on the human gastrointestinal tract, but also animals including pigs, rats, chicken, termites, zebras, and ruminants such as reindeer, sheep, cows, and gazelles have been investigated [1-9]. As is the case with the intestinal ecosystems of many of the carnivore animals, the microbial ecology of the gastrointestinal tract of the polar bear is unknown and we know little about the microbial diversity and dominant species in these animals. The Barents Sea subpopulation of polar bears is located in an area which is sparsely populated by humans and thereby has little contact with human activities [10]. This enables us to study an ecosystem with little human impact.

Antibiotic resistant bacteria are known to originate in populations located in environments that seem not to have been exposed to the selective pressure of pharmaceutically produced antibiotics [11]. The β-lactam antibiotics are of the most widely used agents in clinical and veterinary practice, and resistance to these agents are commonly observed in clinical settings [12]. Some of the most common resistance genes are *bla *genes which encode β-lactamases that give high level resistance to β-lactam antibiotics, and within this group, the *bla*_TEM _genes are very important [13,14]. The *bla*_TEM _alleles encode resistance to ampicillin and other β-lactam antibiotics. Even though widespread in clinical settings, only few studies have determined the distribution of *bla*_TEM _genes in non-clinical environments, included the gastrointestinal tract of free ranging Arctic wild mammals [15-19]. In this study, we have examined the role of polar bear gut microbiota as a potential natural reservoir of the clinically important *bla*_TEM _genes.

Polar bears are major predators in the Arctic marine ecosystem. They are closely associated with sea ice, which they use as substrate for both hunting and movement [20]. The world population of polar bears is currently believed to be about 20,000-25,000 animals that can be divided into 19 subpopulations throughout the circumpolar Arctic [10]. The Barents Sea subpopulation is one of these, and inhabits the geographic regions of Svalbard, the Barents Sea and Franz Josef Land. The size of this subpopulation is estimated to be approximately 2650 individuals [21]. The polar bear has a monogastric digestive system with a simple and relatively short intestine typical of a carnivorous animal, and with the caecum completely lacking [22]. Polar bears are mostly carnivorous and feed mainly on seals, although white whales, narwhals, birds, bird eggs and carrion can be important food items during times of the year when seals are less available [23-30]. In Svalbard, polar bear predation on reindeer on land has also been observed [23].

**Figure 1:**
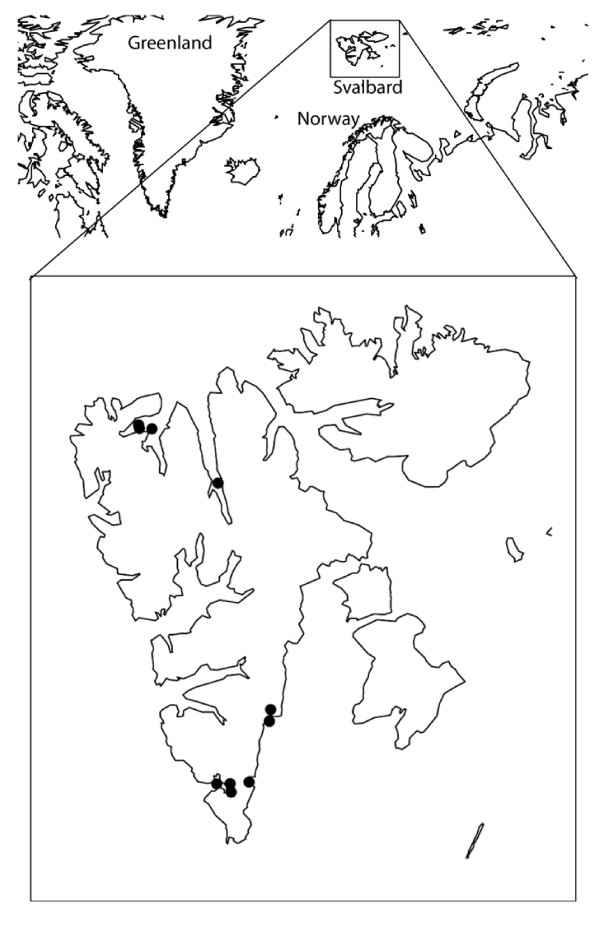
**Map of Svalbard, Norway**. The black circles indicate where the polar bears were captured.

To improve our understanding of the intestinal ecosystem of the polar bear we have studied the bacterial diversity and the prevalence of *bla*_TEM _alleles in faeces of polar bears in Svalbard, Norway (Fig. 1). We here present the results of the molecular characterization of the gastrointestinal microbiota of polar bears sampled through 16S rRNA gene cloning and sequencing.

## Results

### Bacterial diversity

**Figure 2:**
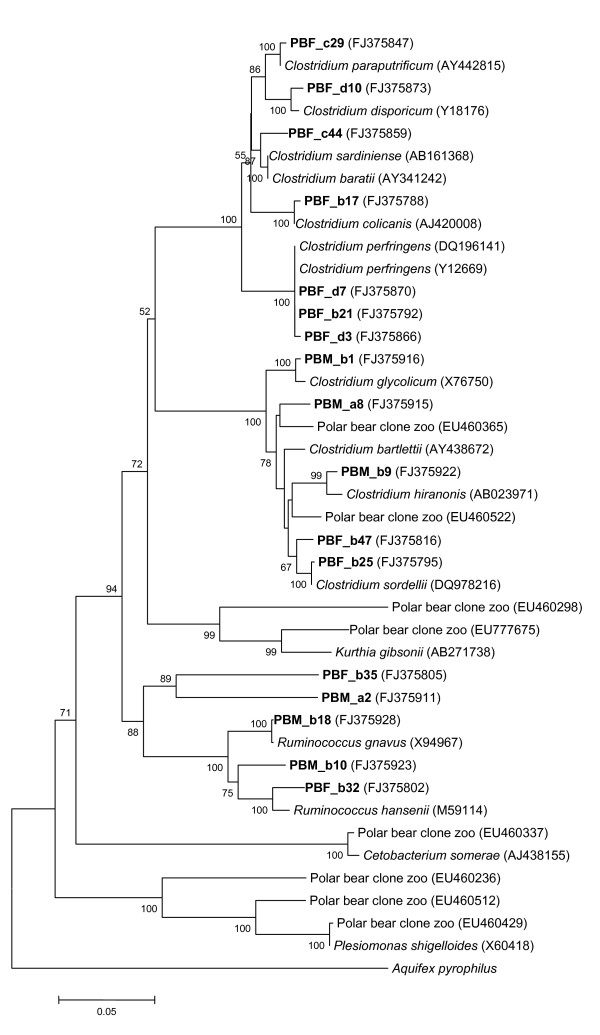
**Phylogenetic tree of the 17 phylotypes recovered from the clone library obtained from faeces from three polar bears in Svalbard, Norway (bold)**. Evolutionary distance was calculated using the Kimura-2 parameter model for nucleotide change and the tree was constructed using the neighbor-joining method. Statistical significance of branching was verified by bootstrapping. The scale bar represents a 5% estimated sequence divergence, and reference sequences were obtained from the GenBank Database.

Sequences were obtained from 161 clones and none of the sequences were identified as possible chimeras. All sequences were affiliated with the phylum *Firmicutes*, with 99% of the sequences belonging to the order *Clostridiales *(Table 1, Fig. 2). The majority of the sequences (70%) were affiliated to the genus *Clostridium*. Based on 97% sequence similarity, seventeen phylotypes were identified (Table 2) within the clone library, with the Chao1 index estimating the population richness to be twenty phylotypes. The Shannon-Weaver index, a measure of diversity, was 1.9, and the coverage was 97%. The most abundant phylotype contained 42% of the sequences, and the nearest relative (99.9%) was *Clostridium perfringens*. Four phylotypes (6% of the sequences) were novel, showing < 97% similarity to sequences representing the phylotypes nearest cultivated relative. Phylotype PBM_a8 contained five sequences and the nearest cultivated relative (96.6%) was *Clostridium bartlettii*. The nearest cultivated relative (95.3%) to phylotype PBF_b32 which contained two sequences was *Ruminococcus hansenii*. The other two phylotypes (PBF_b35 and PBM_a2) contained only one sequence each and the nearest relative belonged to the phylum *Firmicutes *(95.1%) and to unclassified bacteria (96.6%), respectively.

**Table 1:** Distribution and abundance of 16S rRNA gene sequences in the clone library

Organism	No. of clones
Unclassified *Firmicutes*	1
*Clostridiales*	
*Clostridium*	114
*Dorea*	1
*Ruminococcus*	2
*Subdoligranulum*	1
Unclassified *Clostridiaceae*	34
Unclassified *Clostridiales*	8
Total	161

**Table 2:** Polar bear 16S rRNA gene clones representing 17 valid phylotypes

Phylotype	Genbank acc. no.	Size (bp)	No. of clones	Nearest valid relative	Sequence similarity (%)
PBF_d7	FJ375870	1439	67	*Clostridium perfringens *(CP000246)	99.9
PBF_b25	FJ375795	1466	35	*Clostridium sordellii *(DQ978216)	99.5
PBF_c44	FJ375859	1438	18	*Clostridium sardiniense *(AB161368)	98.5
PBM_b9	FJ375922	1427	8	*Clostridium hiranonis *(AB023971)	98.2
PBF_b17	FJ375788	1402	7	*Clostridium colicanis *(AJ420008)	99.8
PBM_b1	FJ375916	1433	5	*Clostridium glycolicum *(X76750)	98.3
PBM_a8	FJ375915	1430	5	*Clostridium bartlettii *(AY438672)	96.6
PBF_c29	FJ375847	1444	3	*Clostridium paraputrificum *(AY442815)	99.7
PBF_b21	FJ375792	1452	2	*Clostridium perfringens *(CP000246)	99.5
PBF_b32	FJ375802	1372	2	*Ruminococcus hansenii *(M59114)	95.3
PBF_b47	FJ375816	1464	2	*Clostridium sordellii *(DQ978216)	98.3
PBM_b18	FJ375928	1459	2	*Ruminococcus gnavus *(X94967)	99.4
PBM_b10	FJ375923	1460	1	*Clostridium sordellii *(DQ978215)	99.5
PBF_d3	FJ375866	1436	1	*Clostridium perfringens *(Y12669)	99.5
PBF_d10	FJ375873	1453	1	*Clostridium disporicum *(Y18176)	98.3
PBF_b35	FJ375805	1488	1	*Firmicutes *bacterium (AF157051)	95.1
PBM_a2	FJ375911	1431	1	Unclassified bacterium (DQ057466)	96.6
Total		161			

### Aerobic heterotrophic cell counts and β-lactamase activity

The aerobic heterotrophic cell counts ranged from 5.0 × 10^4 ^to 1.6 × 10^6 ^cfu/ml for the rectum swabs, and from 4.0 × 10^3 ^to 1.0 × 10^5 ^cfu/g for the faeces samples (Table 3 and 4). The coliform counts for the faeces samples ranged from 3.2 × 10^3 ^to 8.0 × 10^4 ^cfu/g. There was no growth of ampicillin resistant bacteria in the faeces samples. For the rectal swabs, the proportion of amp^r ^bacteria ranged between 3% and 44% (Table 3). A total of 144 randomly selected amp^r ^isolates cultivated from rectal swab samples were tested for β-lactamase activity by the nitrocefin test and all isolates showed β-lactamase activity.

**Table 3:** Aerobic heterotrophic, coliform, and ampicillin resistant cells counts (cfu/ml) in rectum swabs from polar bears in Svalbard

Polar bear no.	Aerobic heterotrophic cells^a^	Amp^r ^aerobic heterotrophic cells^b^	% ^c^
1	5.0 × 10^4 ^(± 5.0 × 10^3^)	1.6 × 10^3 ^(± 6.3 × 10^2^)	3
2	NC	1.0 × 10^4^ (± 1.6 × 10^3^)	-
3	NC	NC	-
5	1.6 × 10^6^(± 2.0 × 10^5^)	8.0 × 10^5 ^(± 1.0 × 10^5^)	44

^a^Mean values are based on nine replicates. ^b^Mean values are based on three replicates. ^c^Percentage of amp^r ^cfu of the total cultivable bacterial population. NC, not possible to count.

**Table 4:** Aerobic heterotrophic, coliform, and ampicillin resistant cell counts (cfu/g) in faeces from polar bears in Svalbard ^a^

Polar bear no.	Aerobic heterotrophic cells	Amp^r ^aerobic heterotrophic cells	Coliform cells	Amp^r ^coliform cells
6	4.0 × 10^3 ^(± 6.3 × 10^2^)	< 11	7.0 × 10^4 ^(± 1.6 × 10^4^)	< 11
7	1.0 × 10^5^(± 1.0 × 10^4^)	< 11	3.2 × 10^3 ^(± 2.0 × 10^3^)	< 11
8 ^b^	8.0 × 10^4 ^(± 1.0 × 10^4^)	< 55	8.0 × 10^4 ^(± 6.3 × 10^3^)	< 55

^a^Values are based on nine replicates. ^b^In the case of polar bear no. 8 only 0.2 gram of faeces sample was taken for subsequent dilution and plating. For all the other animals this amount was 1 gram.

### Detection of *bla*_TEM _genes in amp^r ^isolates

The absence of PCR inhibitory substances in the DNA extracted from amp^r ^isolates was tested by running 16S rRNA gene PCR on extracted DNA from each of 100 single isolates. As much as 98 of the amplifications were positive, indicating that bacterial DNA is amplifiable in 98% of the samples. Subsequently, 144 amp^r ^isolates from the rectal samples were screened for the presence of *bla*_TEM _genes with primers designed for the TEM-1 allele and derivatives [15], and 4 of the amp^r ^isolates were positive. For all four positive isolates, sequencing of the flanking regions demonstrated the presence of *bla*_TEM _inserted in a Tn3 backbone. The four isolates were identified as *E. coli *by ID32 E (bioMérieux, Marcy l'Etoile, France) and 16S rRNA gene sequencing.

### Detection of *bla*_TEM _genes in total genomic DNA extracts

**Figure 3:**
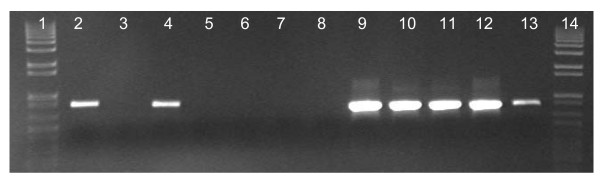
**PCR with *bla*_TEM _specific primers on total DNA extracted from polar bear faeces**. Lane 1 and 14, 1 kb Plus DNA ladder (Invitrogen, California, USA); lane 2, bear no. 6; lanes 3-7, five parallel DNA extraction from faeces from bear no 7; lane 8, bear no. 8; lane 9-13 positive controls (TEM-3, TEM-6, TEM-9, TEM-10, SHV-2).

Total genomic DNA was extracted from the rectal swab from polar bear no. 4 (Table 5). The sample was negative for *bla*_TEM _PCR and positive when screened for 16S rRNA genes, confirming the general suitability of DNA for PCR. Total genomic DNA was also extracted from faeces from three of the polar bears (no. 6-8, Table 5) sampled in 2006, and one of the three faecal samples was negative, while one was positive, and one out of five DNA extractions from the third sample (bear no. 7) was positive (Fig. 3).

**Table 5:** Year of sampling, sex, age, condition, and samples obtained for the polar bear used in this study

Polar bear no.	Sample year	Sex	Age (yrs)	Condition ^a^	Comments	Rectum swab	Faeces sample
1	2004	F	ND	3	Not lactating	X	
2	2004	M	20	3		X	
3	2004	M	22	3		X	
4	2004	M	13	4		X	
5	2004	F	21	4	Not lactating	X	
6	2006	M	2	4	Found together with bear 8		X
7	2006	F	17	4	Found with her 1 year old cub		X
8	2006	M	3	3	Found together with bear 6		X
9 ^b^	2006	M	17	4			X
10 ^b^	2006	M	1	3			X

^a^The animals' conditions are subjectively given values from 1 to 5 to indicate the amount of fat on their bodies, with increasing values indicating more fat; ^b^Samples from polar bears no. 9 and 10 were excluded from further analysis due to low number of cfu/g. ND, not determined.

## Discussion

The Barents Sea subpopulation of polar bears has little contact with human activities [10], and the samples investigated in this study were collected from the subpopulation in their natural environment in Svalbard, Norway. Fresh faeces were collected from live, sedated bears and immediately frozen. There is a potential loss of bacteria when samples are stored before cultivation of bacteria. The pure faeces samples were stored at -70°C and the rectum swabs were stored in 20% glycerol at the same temperature. Achá et al [31] found that there was not a great loss of bacterial number and species when pure faeces samples were stored at -70°C compared to faeces samples mixed with a cryoprotectant such as glycerol, as long as the samples were not repeatedly thawed and analysed in shorter intervals. The samples processed in this study were not repeatedly thawed and analysed and we expect little loss of bacterial number and species compared to if the samples were mixed with glycerol before storing.

The 16S rRNA gene libraries were made from DNA extracted from faeces, and the samples were pooled after PCR to ensure that bacterial DNA from all animals was equally represented. The number of PCR cycles were reduced to a minimum, as the frequency of formation of chimeric molecules increases by the number of PCR cycles [32]. We used 30 cycles for the amplification of the 16S rRNA genes, and did not detect possible chimeras using the Chimera Detection Program. Seventeen different phylotypes were identified among the 161 sequences analysed (Table 2). The coverage of the combined libraries was 97%, which indicate that we have detected the majority of the present microbioma in the faeces. In a study based on faecal microbial communities of 106 individual mammals representing 60 species from 13 taxonomic orders, including captive bears and pandas, Ley et al [33] observed that host diet and phylogeny both influence bacterial diversity, which increases from carnivorous to omnivorous to herbivorous animals. In captive carnivores between 19 and 75 OTUs were observed using the 96% similarity criteria, while in herbivore animals up to 223 OTUs were detected. Within members of the *Ursidae *family including carnivorous, herbivorous and omnivorous bears, the number of OTUs ranged from 14 to 34 which is consistent with our findings.

Only four of the seventeen phylotypes were < 97% related to any known cultivated species (Table 2). This is in contrast to observations made in other studies that the microbial diversity reflected by cultivation represents only a minor fraction of the microbial diversity. In a study of the microbial diversity in reindeer, 92.5% of the bacterial diversity represented novel taxonomic groupings [7]. A study on the microbial community composition of the intestinal tract of chickens, 85% of the phylotypes did not have sequence similarity to any cultured species [1], and in the pig gastrointestinal microbiota, 83% of the identified phylotypes were not likely represented by a known bacterial species [4]. Analysis of the polar bear faeces in this study showed a homogenous microbial flora dominated by Clostridia class. These bacteria are well characterized as they are dominant in the human gut and thereby in the interest of many scientists [34].

All 161 sequences obtained from polar bears were affiliated with the phylum *Firmicutes *(Table 1, Fig. 2). All except one sequence affiliated with the order *Clostridiales*, and 93% to the family *Clostridiaceae*. The low level of diversity observed in the polar bear clone library is in contrast to the diversity observed in colon content from another Arctic carnivorous animal belonging to the same order as polar bears, the hooded seal (*Cystophora cristata*) [35]. Sequences that affiliated with the phyla *Bacteroides*, *Firmicutes*, *Fusobacteria*, and *Proteobacteria *were identified in the colon content from the seals. The dominant phylum was the *Bacteroides *to which 68% of the sequences were affiliated, while 21% were affiliated to the *Firmicutes *[35]. The same molecular methods were used to analyse both the polar bear and seal samples, indicating that the methods are not selective towards *Firmicutes*. Jores et al [36] found *Clostridium *in 44% of the samples when cultivating faeces from polar bears in Svalbard. In faeces from a herbivorous mammal, the wild gorilla, 71% of the phylogenetic lineage was *Firmicutes *[37]. Ley et al [33] observed that the microbial faecal bacterial communities from bears on different diets cluster together, independent of the diet. However, these observations were made in animals kept in zoo's and might not reflect the situation in the wild. Eight of the 673 sequences (GenBank/EMBL/DDBJ database, NCBI) from polar bear faeces collected in zoo's [33] were compared to the sequences obtained in this study (Fig. 2). The eight zoo polar bear sequences included in Fig. 2 represent eight out of 100 phylotypes (analysed by FastgroupII) and contain 59% of the 673 zoo polar bear sequences. Only two of the sequences, representing 10% of all the sequences, cluster together with sequences from our study, indicating a difference between the microbioma in faeces of wild and captive polar bears.

We investigated the prevalence of *bla*_TEM _alleles in faeces from polar bears with little human impact in Svalbard, Norway. We have earlier investigated the prevalence of *bla*_TEM _alleles in Arctic soils and sediments, and in colon content of Arctic seals and found low prevalence of the alleles [15,35]. This current cultivation study of faeces from polar bears did not give any growth on plates with ampicillin (Table 4). The *bla*_TEM _alleles are likely to be found in coliform bacteria, but the selective growth on MacConkey agar with ampicillin yielded < 0.3% amp^r ^cfu (Table 4). However, from 3% to 44% of the isolates from the rectal swabs were phenotypically amp^r^. A random selection of amp^r ^isolates all showed β-lactamase activity, but when tested by *bla*_TEM _PCR, only 4 out of 144 isolates were positive. This indicates a low level of *bla*_TEM _alleles. The four isolates were all identified as *E. coli*, and the *bla*_TEM _alleles were inserted in a Tn3 transposon which is found in a wide variety of bacteria. The presence of *bla*_TEM _alleles has previously been reported in wild animals in Portugal, where they detected the alleles in *E. coli *isolated from faeces from deer, fox, owl, and birds of prey [38]. Others have identified *bla*_TEM _in faecal *E. coli *isolates from pigs, dogs, and cats [17,39]. The *bla*_TEM _PCR on total DNA extracted was negative for the two rectal swabs, and two of the three faecal samples were *bla*_TEM _PCR positive (Fig. 3). Previous studies on Arctic soil samples suggest that the detection limit for total DNA extracted was < 21 *bla*_TEM _alleles (pUC18) per PCR sample [15]. The diversity analysis of polar bear faeces showed a dominance of clostridiales in which there has been no reports of β-lactamase production. This is consistent with the low levels of *bla*_TEM _alleles detected in the samples.

## Conclusions

This study showed that the bacterial diversity in faeces from polar bears in their natural environment in the pristine Svalbard area were low, all obtained clones affiliated to Firmicutes. As with any PCR-based method, 16S rRNA gene clone libraries are biased [40] and the gastrointestinal microbiota of more polar bears should be studied to give a more complete picture of the microbial diversity. Furthermore, only low levels of *bla*_TEM _alleles were detected in contrast to their increasing prevalence in some clinical and commensal bacterial populations.

## Methods

### Sampling

Ten samples from ten polar bears were collected on two occasions. Faeces were sampled from five individuals March 30^th^-April 12^th ^2004 and from five individuals March 30^th^-April 9^th ^2006 (Table 5). Sampling occurred on both occasions at the coast or the surrounding sea ice at Spitsbergen and Nordaustlandet in Svalbard, Norway (Fig. 1). Bears were caught by remote injection of a dart (Palmer Cap-Chur Equipment) containing the drug Zoletil^® ^(Virbac, Carros Cedex, France) fired from a helicopter [41]. Animal handling methods were approved by the National Animal Research Authority (Norwegian Animal Health Authority, P.O. Box 8147 Dep., N-0033 Oslo, Norway). The sex, reproductive status, and a series of standardized morphometric measurements were collected from each bear (Table 5). In 2004, the samples were collected by swabbing rectum and the samples were kept frozen in LB-broth (Luria Broth, Fluka BioChemica) with 20% glycerol. In 2006, faeces was collected with a sterile glove and kept in sterilized plastic bags. The amount of sample ranged from 0.2 g to 2 g. All samples were kept in containers at -20°C during transport to the laboratory, where they were stored at -70°C until analysed.

### 16S rRNA Clone Library

The amount of sampled material was limited due to little faeces in the rectum of the polar bears, and only three faeces samples gave sufficient DNA yield to make 16S rRNA gene clone libraries. A 16S rRNA gene clone library was made with DNA extracted from faeces from bear no. 6, 7 and 8. Total genomic DNA was extracted using the QIAmp DNA stool kit (Qiagen, Solna, Sweden) according to the protocol provided by the producer, and DNA quantified using a NanoDrop^® ^ND-1000 Spectrophotometer (260 nm) (Thermo Fisher Scientific, Waltham, USA). Two parallel 16S rRNA gene PCR amplifications on DNA from each of the three animals were performed, using primers 16S-27F and 16S-1494R (Table 6), in a reaction mixture containing 1× HotStartTaq DNA master mix (Qiagen), 0.3 μM of each primer, and 20 ng of extracted DNA solution in a final volume of 50 μl. PCR amplification was initiated by denaturation at 95°C for 15 min and then 30 cycles of 94°C for 30 s, 50°C for 30 s, and 72°C for 2 min, with a final extension at 72°C for 10 min. The 16S rRNA gene amplicons were pooled and cloned using the TOPO TA Cloning^® ^Kit for Sequencing (Invitrogen, California, USA), and transformed by heat-shock into One Shot^® ^Competent *Escherichia coli *cells (Invitrogen). Positive clones were randomly selected and recombinant plasmids extracted using QIA prep spin miniprep kit (Qiagen). Extracted DNA was quantified using a NanoDrop ND-1000 Spectrophotometer (260 nm), and sequenced on a 3130 Genetic analyzer (Applied Biosystems, Foster City, USA) using the ABI BigDye Terminator chemistry. The sequencing primers (Invitrogen) used were M13 forward primer, M13 reverse primer, and the universal bacterial 16S rRNA primer Bact338, corresponding to nucleotide position 338-355 of *E. coli *(Table 6).

**Table 6:** Primers used for PCR and sequencing

Name	Primer sequence (5'-3')	Gene target	Reference
BlaF	CATTTCCGTGTCGCCCTTATTCC	*bla*_TEM_	[52]
BlaR	GGCACCTATCTCAGCGATCTGTCTA	*bla*_TEM_	[52]
TemI3	TGGTTTATTGCTGATAAATCTGGAG	*bla*_TEM_	[15]
TemI5a	TTAAAAGTGCTCATCATTGGAAAAC	*bla*_TEM_	[15]
TemI5b	CTGTTGAGATCCAGTTCGATGTA	*bla*_TEM_	[15]
16S-27F	AGAGTTTGATCCTGGCTCAG	16S rRNA	[53]
16S-1494R	CTACGGCTACCTTGTTACGA	16S rRNA	[53]
Bact338	GCTGCCTCCCGTAGGAGT	16S rRNA	[54]

### Sequence analysis

The 16S rRNA gene sequences were assembled using the program Lasergene™ Seqman v. 7.1.0. (DNASTAR Inc.). Putative chimeric sequences were evaluated using the Chimera Detection Program which is part of the SimRank 2.7 package available through the Ribosomal Database Project (RDP) [42]. Sequences generated were first compared to sequences obtained from the RDP II (Classifier: Naive Bayesian rRNA Classifier Version 1.0, November 2003; The nomenclature taxonomy of Garrity and Lilburn, release 6.0) and then compared to GenBank sequences using BLAST (Basic Local Alignment Search Tool) [43]. The 16S rRNA gene sequences were automatically aligned by CLUSTAL-W in the software package BioEdit (v. 5.0.9) to give a uniform length. Phylogenetic analysis was performed using the neighbour-joining method with the Kimura2-parameter correction model in the software MEGA (v. 4.0) [44]. Statistical significance of branching was verified by bootstrapping [45] involving construction and analysis of 1000 trees from the data set in the software MEGA. Sequences were assigned to operational taxonomic units (OTUs) based on a 97% sequence similarity criterion [46]. Standard diversity and richness indices, including the Shannon-Weaver index [47] (a nonparametric diversity index combining estimates of richness, i.e. total numbers of ribotypes) and evenness (relative abundance of each OTU, indicating diversity) and the Chao1 index [48] (a nonparametric estimator of the minimum OTU richness) were calculated using the FastGroupII web-based bioinformatics platform for analyses of 16S rRNA gene based libraries [49]. The coverage of the clone library was calculated with the formula [1-(n/N)] [50] where n is the number of phylotypes (OTUs) represented by one clone and N is the total number of clones. The sequence data for the clones have been submitted to the GenBank/EMBL/DDBJ database (NCBI) with accession numbers FJ375772 to FJ375932.

### Determination of cultivable, coliform, and ampicillin resistant counts

Faeces samples were thawed and suspended in saline immediately before cultivation of aerobic bacteria. For both rectal swabs and faeces samples, colony forming units were determined for aerobic heterotrophic cells on chocolate medium (agar, horse blood, glucose, Vitox SR 090A, Vitox, SR 090H (Oxoid); University hospital, Tromsø, Norway) and for amp^r ^aerobic heterotrophic cells on chocolate medium supplemented with 50 mg/l of ampicillin (Sigma). Coliform cells were determined for faeces samples on MacConkey medium (Fluka BioChemika), and for amp^r ^coliform cells on MacConkey medium supplemented with 50 mg/l of ampicillin. All plates were enumerated after 48 h of incubation at 37°C. Means and standard deviations (SD) for the cfu's were calculated on the basis of nine replicates for each of the bear samples analysed.

### Identification of β-lactamase activity with the nitrocefin-test

Extracellular β-lactamase activity was determined by the nitrocefin test method. A solution (0.5 g/l) of nitrocefin (chromogenic β-lactamase substrate, Calbiochem, San Diego, USA) was prepared according to the manufacturer's instruction. Ten μl of the solution was added to single colonies and a colour change from yellow to pink within 30 minutes after application indicated β-lactamase activity.

### DNA extraction and test PCR amplification of 16S rRNA genes

DNA was extracted from randomly chosen colonies by a boiling lysis method [51]. The general suitability of DNA for PCR was confirmed with amplification of the 16S rRNA gene, using the primers 16S-27F and 16S-1494R (Table 6). The amplification was performed as explained above, with the following conditions; denaturation at 95°C for 15 min and then 5 cycles of 94°C for 4 min, 50°C for 45 s, and 72°C for 1 min, and then 30 cycles of 92°C for 45 s, 55°C for 45 s, and 72°C for 1 min, with a final extension at 72°C for 10 min.

### PCR amplification of potential *bla*_TEM _genes in amp^r ^isolates

The amplification of *bla*_TEM _alleles in individual bacterial isolates was performed in a reaction mixture containing 1× HotStartTaq DNA master mix (Qiagen), 0.2 μM of each primer, and 2 μl of the crude DNA solution in a final volume of 30 μl. Reactions were denatured at 95°C for 15 min and then subjected to 30 cycles of 94°C for 45 s, 61°C for 45 s, and 72°C for 1 min, with a final extension at 72°C for 10 min. For all *bla*_TEM _PCR analyses, the primers BlaF and BlaR (Table 6) were used to amplify a product of 828 bp (TEM-1 allele of *E. coli*) [15]. The following controls were used: five strains of *E. coli *carrying the *bla *alleles TEM-1, TEM-3, TEM-6, TEM-9, and TEM-10 as positive controls, and one strain carrying the SHV-2 allele as negative control. The specificity of the primers were confirmed by 'in silico' amplification and by aligning the primer binding region of approximately 100 sequence polymorphic *bla*_TEM _alleles [15].

### Sequencing of 16S rRNA, *bla*_TEM_, and *bla*_TEM _flanking regions

The identity of putative amp^r ^positive isolates was determined by sequencing, with primers 16S-27F, 16S-1494R, and Bact 338 (Table 6), on a 3130 Genetic analyzer using the ABI BigDye Terminator chemistry. To confirm the presence of and determine the location of *bla*_TEM _in the DNA extract from amp^r ^isolates, sequencing of the immediate flanking regions of the *bla*_TEM _gene was performed using the sequencing primers TemI3, TemI5a or TemI5b (Table 6) as described in [15].

## Authors' contributions

TG has participated in its design and coordination, participated in the analysis, and drafting and revising the manuscript. MAS conceived part of the study, participated in its design and analysis, and revising the manuscript. KN conceived part of the study, participated in its design and revision of the manuscript. PB performed molecular genetic analyses/cultivations and drafting of the manuscript. LB has participated in the analysis and interpretation of data, and revising the manuscript. JA has been involved in acquisition of data and revising the manuscript. MA has been involved in acquisition of data and revising the manuscript. All authors read and approved the final manuscript.
